# A potent and durable malaria transmission-blocking vaccine designed from a single-component 60-copy Pfs230D1 nanoparticle

**DOI:** 10.1038/s41541-023-00709-8

**Published:** 2023-08-18

**Authors:** Nichole D. Salinas, Rui Ma, Thayne H. Dickey, Holly McAleese, Tarik Ouahes, Carole A. Long, Kazutoyo Miura, Lynn E. Lambert, Niraj H. Tolia

**Affiliations:** 1 Host-Pathogen Interactions and Structural Vaccinology Section, Laboratory of Malaria Immunology and Vaccinology, National Institute of Allergy and Infectious Diseases, National Institutes of Health, Bethesda, MD USA; 2 Vaccine Development Unit, Laboratory of Malaria Immunology and Vaccinology, National Institute of Allergy and Infectious Diseases, National Institutes of Health, Bethesda, MD USA; 3 Laboratory of Malaria and Vector Research, National Institute of Allergy and Infectious Diseases, National Institutes of Health, Rockville, MD USA

**Keywords:** Protein vaccines, Adjuvants, Malaria

## Abstract

Malaria transmission-blocking vaccines (TBVs) reduce disease transmission by breaking the continuous cycle of infection between the human host and the mosquito vector. Domain 1 (D1) of Pfs230 is a leading TBV candidate and comprises the majority of transmission-reducing activity (TRA) elicited by Pfs230. Here we show that the fusion of Pfs230D1 to a 60-copy multimer of the catalytic domain of dihydrolipoyl acetyltransferase protein (E2p) results in a single-component nanoparticle composed of 60 copies of the fusion protein with high stability, homogeneity, and production yields. The nanoparticle presents a potent human transmission-blocking epitope within Pfs230D1, indicating the antigen is correctly oriented on the surface of the nanoparticle. Two vaccinations of New Zealand White rabbits with the Pfs230D1 nanoparticle elicited a potent and durable antibody response with high TRA when formulated in two distinct adjuvants suitable for translation to human use. This single-component nanoparticle vaccine may play a key role in malaria control and has the potential to improve production pipelines and the cost of manufacturing of a potent and durable TBV.

## Introduction

There were an estimated 247 million cases of malaria and 619,000 deaths as a result of those infections in 2021^1^. Malaria is caused by *Plasmodium* parasites that have a life cycle consisting of the pre-erythrocytic and asexual/erythrocytic stages in the human host and a sexual reproduction stage that occurs in the mosquito vector^2,3^. Male and female *Plasmodium* gametocytes from an infected human are taken up by a mosquito during a blood meal resulting in parasite transmission from one infected human to another by the mosquito. The gametocytes within the mosquito midgut rapidly differentiate into gametes that can undergo sexual fertilization to form zygotes. The zygotes further develop into ookinetes, followed by oocysts, and finally, sporozoites that migrate to the salivary glands of the mosquito. These sporozoites are available for injection into the next human host upon the next mosquito blood meal and begin the malaria disease cycle anew.

The complexity of the life cycle makes developing effective interventions by either drugs or vaccines difficult, and the production of new control measures is critical as parasite resistance to front-line drug therapies and mosquito resistance to pesticides is on the rise^4^. A key strategy for malaria vaccine development is to prevent disease transmission by transmission-blocking vaccines (TBVs). Pfs230 is the leading TBV candidate under evaluation in current clinical trials (NCT02334462, NCT02942277, NCT03917654, NCT05135273). Pfs230 TBVs target parasite development within the mosquito midgut to disrupt the cycle of infection.

**Fig. 1:**
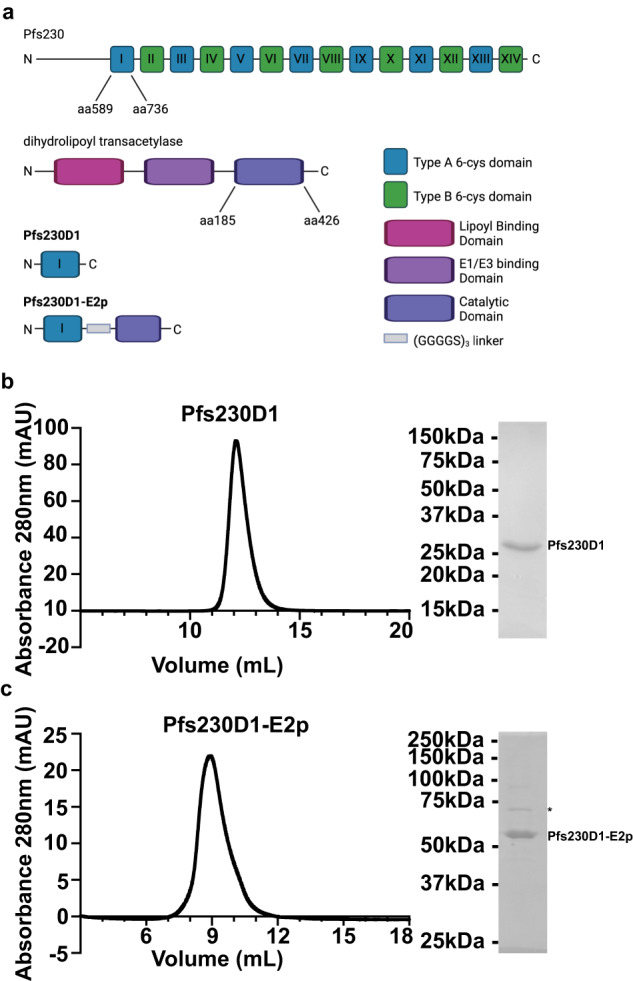
Pfs230D1 and Pfs230D1-E2p can be expressed in Expi293F cells. **a** Domain architecture of Pfs230 and E2p full length proteins and Pfs230D1 and Pfs230D1-E2p constructs. Created with BioRender.com. Evaluation of size and purity of **b** Pfs230D1 and **c** Pfs230D1-E2p by size-exclusion chromatography (left) and reduced SDS-PAGE gel electrophoresis (right). The minor contaminant band observed in the SDS-PAGE analysis (*) was identified by mass spectrometry as heat shock protein (HSP) 70–1B. The molecular weight marker for the SDS-PAGE gel electrophoresis in (**b**) and (**c**) corresponds to the BIO-RAD Precision Plus Protein Dual Color Standards.

Pfs230^5–8^ is a member of the 6-cys family of proteins specific to *Plasmodium* parasites. Members of the 6-cys family all contain domains with conserved cysteine residues and are expressed in the sexual and asexual stages. Pfs230 is a 363 kDa protein expressed in gametocytes and gametes that undergoes proteolytic processing upon gametogenesis^9–11^. The processed Pfs230 is approximately 300 kDa and plays a role in the fertilization of female macrogametes by the male microgametes^9,12,13^. Pfs230 is anchored to the gametocyte/gamete membrane through interactions with Pfs48/45^9,12–14^. The processed protein consists of an N-terminal region followed by fourteen 6-cysteine domains^7,15^ (Fig. 1a).

Individual domains and larger multidomain segments have been examined as vaccine immunogens and domain 1 (D1) and a portion of the unstructured N-terminal region elicit antibodies with transmission-reducing activity (TRA) or transmission-blocking activity (TBA)^6,7,16–21^. D1 conjugation to large carrier protein complexes like ExoProtein A (EPA)^22–26^, incorporation into virus-like particles (VLPs)^21,27^, or conjugation to liposomes^28,29^ or outer membrane vesicles^24^ greatly increased the TBA or TRA induced. Alternate methods for nanoparticle formation include two-component nanoparticles^21–27,30^ and tag coupling systems like SpyTag/SpyCatcher^31–35^. Both these systems separate the antigen from the multimeric component and allow for nanoparticle assembly in vitro prior to immunization^21–27,30–35^. The increase in TRA induced by these systems is consistent with the principle that incorporation of proteins into larger nanoparticles improves the immune response to smaller antigens through multiple mechanisms, including repetitive display, increased uptake by antigen-presenting cells and increased activation of T-helper cells^30^.

The generation of the nanoparticles listed above all share one weakness which is that they all require multiple steps to produce the final product. Conjugation requires the production of multiple individual components and a subsequent chemical conjugation step^22^. The production of the VLPs requires the co-production of the unmodified nanoparticle core with the fusion protein to create the VLP^21,27^. Conversely, a single-component nanoparticle with high potency and longevity has the potential to simplify production and reduce cost. Single-component nanoparticle vaccines have recently been developed for diverse diseases and have progressed to clinical trials for influenza^36^ (NCT03186781 and NCT03814720) and HIV^37^ (NCT03547245). One single-component nanoparticle system for vaccine design is the catalytic domain of dihydrolipoyl acetyltransferase protein (E2p) of the 2-oxo acid dehydrogenase multienzyme complex from *Geobacillus stearothermophilus* (formerly *Bacillus stearothermophilus*)^38,39^.

The 2-oxo acid dehydrogenase multienzyme complex or pyruvate dehydrogenase complex (PDH complex) is a conserved complex that is responsible for the oxidative decarboxylation of pyruvate to acetyl-CoA, an essential molecule for multiple metabolic pathways, including the Krebs cycle^40–55^. The PDH complex consists of three enzymes that each form homo-oligomers: pyruvate dehydrogenase (E1), dihydrolipoyl acetyltransferase (E2), and dihydrolipoamide dehydrogenase (E3). The E2 protein forms the core of the PHD complex and is composed of three domains: lipoyl binding domain, E1/E3 binding domain, and catalytic domain (Fig. 1a). The E2 protein can form homo-trimers that assemble into either a 24-copy cube with octahedral symmetry^40–44^ or a 60-copy pentagonal dodecahedron core with icosahedral symmetry^45–53^ depending on the species. The E2 protein from gram-negative bacteria, like *E. coli*, assemble into the 24-copy cube^40–44^, while gram-positive bacteria, like *G. stearothermophilus*^45–49^, yeast, and mammalian organisms form the 60-copy pentagonal dodecahedron^50–53^.

The E2 protein from *G. stearothermophilus* first assembles into homo-trimers that then assemble into the 60-copy pentagonal dodecahedron with 532-fold icosahedral symmetry^45–49^. The interface of the homo-trimers creates the catalytic site for the acetyltransferase. Truncated E2 proteins that only contain the catalytic domain are still able to form the 60-copy pentagonal dodecahedral in the absence of the lipoyl binding and E1/E3 binding domains^46^. The N-terminus of these truncated proteins is available for the fusion of antigens and has been used successfully to present peptides from *P. falciparum* circumsporozoite protein (CSP)^39^, trimeric gp120 from HIV-1^38^, and a modified Receptor Binding Domain (RBD) of the SARS-CoV2 spike protein called noNAG-RBD-E2p^56^. Both the CSP-E2p fusion^39^ and the noNAG-RBD-E2p fusion were immunogenic^56^, and noNAG-RBD-E2p elicited neutralizing antibodies that recognized and neutralized SARS-CoV-2 WA-1 and variants^56^. The large size and cage-like structure of the E2 catalytic domain allow for the attachment of antigens with diverse sizes and oligomeric states^30,39^ to enhance the immune response upon vaccination.

Repetitive display of antigens on nanoparticles is one way to enhance the immune response to a vaccine. It is also critical to optimize the adjuvant used in conjunction with the antigen. Several clinically approved adjuvants include aluminum hydroxide, aluminum phosphate, MF59, AS01, AS03, AS04, and CpG 1018, and each has unique immunostimulatory mechanisms^57–61^. The adjuvant Alhydrogel is a wet gel suspension of aluminum hydroxide that is known to induce antibody responses independent of Toll-like receptor signaling and cause the activation of macrophages and dendritic cells^57,58,60,61^. A second distinct class of adjuvants is squalene-based oil-in-water emulsions that include AS03 and the research-grade equivalent AddaS03. AS03/AddaS03 can induce cytokine and chemokine responses at the site of injection and in draining lymph nodes resulting in enhanced antigen uptake by monocytes^57,59,61^. Both Alhydrogel and AddaS03 are commercially available and can be readily applied to the development of new vaccines.

Here we show that a Pfs230D1-E2p fusion protein can be stably expressed in a mammalian expression system. The fusion protein forms a stable, homogenous 60-copy nanoparticle that can be recognized by a Pfs230D1-specific transmission-blocking monoclonal antibody with a conformation-dependent epitope indicating a successful display of Pfs230D1. We show that Pfs230D1-E2p produces a potent and durable antibody response, particularly when formulated in adjuvants suitable for translation to human use and that are readily sourced or produced. These results provide the basis for an enhanced transmission-blocking vaccine composed of an easily manufactured single-component antigen and human-applicable adjuvants.

## Results

### Pfs230D1 and Pfs230D1-E2p can be expressed and purified from mammalian cells

While domain 1 (D1) of Pfs230 has previously been expressed in several different systems, the catalytic domain of dihydrolipoyl acetyltransferase protein (E2p) of the 2-oxo acid dehydrogenase multienzyme complex from *G. stearothermophilus* has been primarily expressed in bacterial systems, such as *E. coli*, which are likely incompatible with formation of disulfide bridging critical for proper folding of Pfs230D1. To find an expression system that could allow for the expression of Pfs230D1 and Pfs230D1-E2p nanoparticles, we tested the expression of Pfs230D1 and Pfs230D1-E2p in the Expi293F cell mammalian culture system. Pfs230D1 and Pfs230D1-E2p pHLSec plasmids were generated for the expression of both constructs in mammalian cell culture.

Transient transfection of Expi293F cells with Pfs230D1 resulted in high levels of protein in the supernatant of transfected cultures four days post-transfection. Pfs230D1 was purified from the supernatant using Ni2+ resin followed by size-exclusion chromatography (Fig. 1b). The Pfs230D1 monomer elutes from a Superdex 75 10/300 increase size-exclusion column (Cytiva) at 12 mL and a single band of approximately 25 kDa can be seen on a reducing SDS-PAGE gel. This is consistent with the theoretical molecular weight of 22.9 kDa. Further analysis of the purified protein by size-exclusion chromatography small angle X-ray scattering (SEC-SAXS) and multi-angle light scattering (SEC-MALS) reported molecular weights of 27.5 kDa and 21.8 kDa ± 0.1 kDa, respectively, (Table 1) consistent with the mass of a monomer.

**Table 1:** Molecular weight of Pfs230D1 and Pfs230D1-E2p expressed in Expi293F cells.

	Subunit theoretical molecular weight (kDa)	Theoretical oligomeric state	Particle theoretical molecular weight (kDa)	Molecular weight determined by SEC-SAXS (kDa)	Molecular weight determined by SEC-MALS (kDa)	R_g_ determined by SEC-SAXS (Å)
Pfs230D1	22.9	Monomer	---	27.5	21.8 ± 0.1	25.80 ± 0.38
Pfs230D1-E2p	49.4	60-copy	2966	2903.6	3447 ± 13	130.47 ± 11

The theoretical molecular weights for monomeric proteins were calculated using the Expasy ProtParam tool and the molecular weight of the 60-copy Pfs230D1-E2p nanoparticle was calculated by multiplying the Pfs230D1-E2p fusion monomer molecular weight by 60. The experimentally determined molecular weight from SEC-SAXS data was determined by analyzing SEC-SAXS data in PRIMUS from the ATSAS software suite. The experimentally determined molecular weight from SEC-MALS data using Wyatt Astra 6 software and the Mw value and standard deviation reported. The radius of gyration (R_g_) with standard deviation was determined by analyzing SEC-SAXS data in PRIMUS from the ATSAS software suite.

Transient transfection of Expi293F cells with Pfs230D1-E2p plasmid also resulted in protein in the supernatant of transfected cultures 4 days post-transfection. Pfs230D1-E2p was further purified by size-exclusion chromatography of the concentrated supernatant in a tag-less purification scheme. The theoretical molecular weight of a single Pfs230D1-E2p fusion protein is 49.4 kDa (Table 1). Previous studies have shown that E2p forms a 60-copy nanoparticle^45–49^ and the Pfs230D1-E2p nanoparticle would therefore have a theoretical molecular weight of 2966 kDa (Table 1). An SRT SEC-1000 column (Sepax Technologies) was used for the size-exclusion chromatography, given the expected large size of the nanoparticle. The Pfs230D1-E2p nanoparticle eluted from the Sepax SRT SEC-1000 column at 9 mL, consistent with the formation of a nanoparticle, and an approximately 50 kDa band can be seen on a reducing SDS-PAGE gel consistent with the theoretical molecular weight of the monomer (Fig. 1c). The molecular weight determined by SEC-SAXS was 2903.6 kDa and by SEC-MALS was 3447 kDa ± 13 kDa (Table 1). These molecular weights are consistent with the theoretical molecular weight of the 60-copy nanoparticle expected for the E2p nanoparticle^39,45,46,48^.

### Pfs230D1-E2p forms a uniform 60-copy nanoparticle

**Fig. 2:**
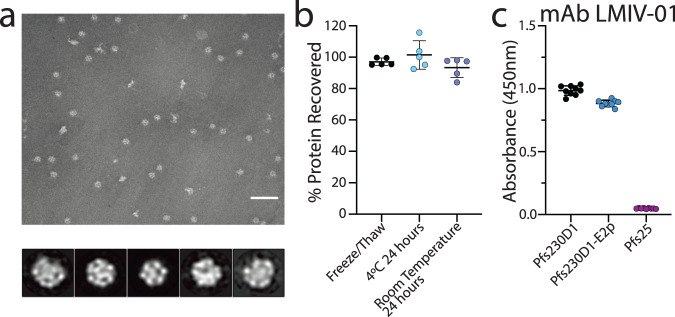
Pfs23D1-E2p forms uniform nanoparticles that are stable and recognized by LMIV-01, a Pfs230D1-specific monoclonal antibody. **a** (Top) Negative-stain electron microscopy image of Pfs230D1-E2p image (top) scale bar = 100 nm and 2D class averages of the particles (bottom). **b** Percent recovery of purified Pfs230D1-E2p after being exposed to freeze/thaw, room temperature for 24 h, and 4 °C for 24 h. Each point represents a paired biological replicate of initial time zero purification and a purification for each condition. Mean and standard deviation are shown. **c** Binding of LMIV-01 monoclonal antibody to Pfs230D1, Pfs230D1-E2p, and Pfs25 by ELISA. Each point is a biological replicate with three technical replicates. Mean and standard deviation are shown.

We conducted negative-stain electron microscopy of Pfs230D1-E2p to examine sample quality. Negative-stain images showed highly homogenous samples, and the 2D class averages clearly indicate that the purified protein assembles as nanoparticles (Fig. 2a). The negative-stain images and the 2D class averages are consistent with the 60-copy structure E2p of *G. stearothermophilus*^45,48,49^. In summary, the SEC elution volume, the SEC-SAXS and SEC-MALS calculated masses, and the size of the particles by negative-stain electron microscopy are all consistent with Pfs230D1-E2p forming a 60-copy nanoparticle (Figs. 1c and 2a).

### Pfs230D1-E2p is stable in solution under multiple conditions

An important characteristic of potent vaccines is that they are stable over time and under various conditions. To determine the stability of our nanoparticle, we exposed the nanoparticle to a single cycle of freeze/thaw to 24 h at 4 °C and to 24 h at room temperature. For each condition, a biological replicate was split equally between a time zero no treatment sample and a treatment sample. The particle integrity of the time zero treatment was immediately analyzed by size-exclusion chromatography, and the treatment sample was analyzed by size-exclusion chromatography after the given treatment conditions had been applied. The nanoparticle was stable under all conditions tested, as shown by a percent recovery of protein that was in the range 95.2–99.8% for freeze/thaw, 92.4–115.7% for 24 h at 4 °C, and 84–98.1% for 24 h at room temperature (Fig. 2b and Supplementary Fig. 1).

### The Pfs230D1-E2p nanoparticles present a potent human transmission-blocking epitope

We examined whether the correct transmission-blocking surfaces of Pfs230D1 are exposed and available in the nanoparticle by evaluating the binding of LMIV-01, a Pfs230D1-specific human antibody with potent transmission-reducing activity (TRA). LMIV-01 is a human monoclonal antibody isolated from an individual that was immunized with Pfs230D1-EPA conjugate^6^ that binds to Pfs230 on gametocyte stage parasites and has potent TRA in a standard membrane feeding assay (SMFA)^6^. Binding of LMIV-01 to Pfs230D1, Pfs230D1-E2p, and Pfs25^3,62–64^, another *Plasmodium* gametocyte stage protein of similar size to Pfs230D1, was evaluated by enzyme-linked immunosorbent assay (ELISA) (Fig. 2c). LMIV-01 binds to both Pfs230D1 and Pfs230D1-E2p but not to Pfs25, which demonstrates that Pfs230D1 presents the functional epitope when tethered on the surface of the nanoparticle.

### A two-vaccination regimen for Pfs230D1-E2p is sufficient to produce high antibody titers to Pfs230D1

**Fig. 3:**
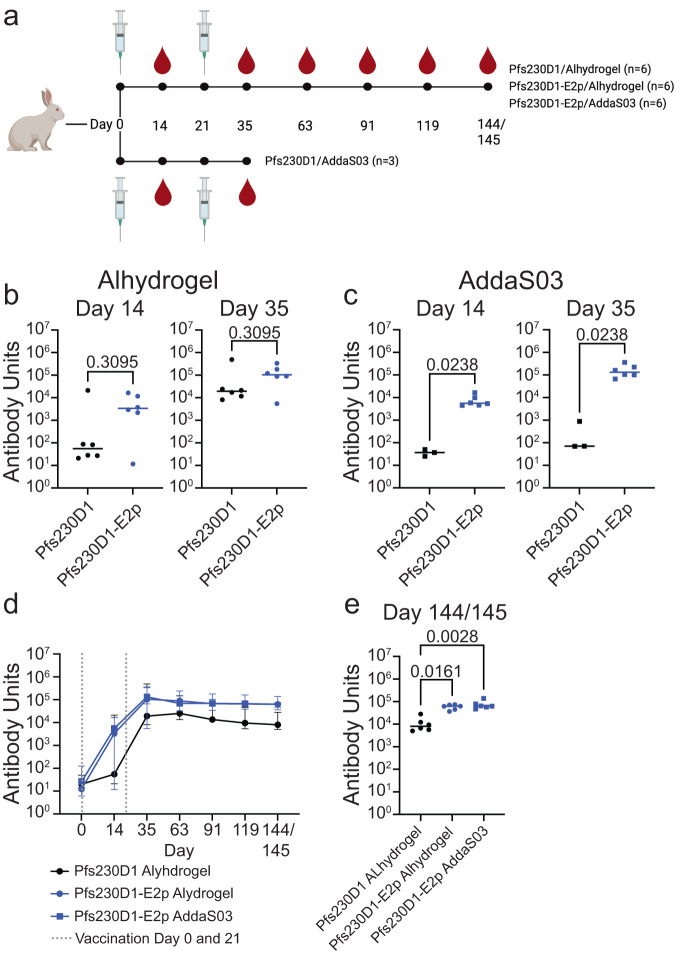
Pfs230D1-E2p induces long-lived high levels of Pfs230D1-specific antibodies. **a** Timeline and groups for rabbit immunizations. Created with BioRender.com. **b** Day 14 and 35 Pfs230D1-specific antibody titers after one and two immunizations, respectively, of rabbits with 1 μg of either Pfs230D1 or Pfs230D1-E2p formulated with Alhydrogel on days 0 and 21. *p*-values displayed were determined using a Mann–Whitney test with all data and not eliminating any outliers, and bars represent the median. Mann–Whitney analysis with the elimination of the single outliers from each group resulted in *p*-values of 0.0079 and 0.0079 on day 14 and day 35, respectively. **c** Day 14 and 35 Pfs230D1-specific antibody titers after one and two immunizations, respectively, of rabbits with 1 μg of either Pfs230-D1 or Pfs230D1-E2p formulated with AddaS03 on days 0 and 21. The *p*-values were determined using a Mann–Whitney test, and the bars represent the median. **d** Median Pfs230D1-specific antibody titers for rabbits immunized with Pfs230D1/Alhydrogel, Pfs230D1-E2p/Alhydrogel, and Pfs230D1-E2p/AddaS03 over time. The error bars represent the range between the highest and lowest values. **e** Pfs230D1-specific antibody titers on day 144 (Pfs230D1/Alhydrogel) and day 145 (Pfs230D1-E2p/Alhydrogel and Pfs230D1-E2p/AddaS03) for rabbits immunized with Pfs230D1/Alhydrogel, Pfs230D1-E2p/Alhydrogel, and Pfs230D1-E2p/AddaS03. The *p*-values were calculated using a Kruskal–Wallis test with Dunn’s multiple comparisons and bars represent the median.

Female New Zealand White rabbits were immunized on day 0 and day 21 with 1 μg of either Pfs230D1 or Pfs230D1-E2p formulated in either Alhydrogel or AddaS03 as described in “Methods” (Fig. 3a). Alhydrogel is an aluminum hydroxide-based adjuvant that enhances immune responses and often results in an IgG1 dominant antibody response^60,61^. In contrast, AddaS03, the research-grade equivalent of AS03, is an oil-in-water emulsion adjuvant that has a wider, more balanced antibody response^57,59,61^. These adjuvants were selected as they are suitable for translation to human use, easily sourced or produced in large quantities, and have distinct immune response profiles.

Antibody responses were evaluated 2 weeks post-vaccination on day 14 and day 35 by Pfs230D1-specific ELISA. Animals immunized with Pfs230D1-E2p formulated with Alhydrogel had higher antibody titers than those immunized with Pfs230D1 monomer in Alhydrogel, although the difference was not statistically significant at either time point (*p*-value = 0.3095 at both day 14 and day 35) (Fig. 3b). Analysis of the *p*-values was conducted using all data, although both groups each contain one outlier. Animals immunized with Pfs230D1-E2p formulated in AddaS03 had significantly higher titers than animals immunized with Pfs230D1 monomer formulated in AddaS03 at both day 14 and day 35 (*p*-value = 0.0238) (Fig. 3c). The one animal in the Pfs230D1-E2p/AddaS03 group that had a lower titer than the others did increase in titers by day 63 and had similar levels to the other animals in that group for all subsequent time points.

### Pfs230D1-E2p elicits a durable antibody response

We evaluated the longevity of the antibody response upon immunization with the various vaccine formulations developed. Animals immunized with Pfs230D1/Alhydrogel, Pfs230D1-E2p/Alhydrogel, and Pfs230D1-E2p/AddaS03 were evaluated every 28 days up to day 144 (Pfs230D1/Alhydrogel) and day 145 (Pfs230D1-E2p/Alhydrogel and Pfs230D1-E2p/AddaS03). Pfs230D1/AddaS03 animals were not followed as they did not have high titers on day 35. Pfs230D1-specific antibody titers after two immunizations were lower for Pfs230D1/Alhydrogel as compared to either Pfs230D1-E2p/Alhydrogel or Pfs230D1-E2p/AddaS03 and remained lower over this time period (Fig. 3d). The difference between the monomer titers and the nanoparticle titers also increases slightly over time. For example, at day 35, Pfs230D1-E2p/Alhydrogel and Pfs230D1-E2p/addaS03 elicit GMTs of 82,402 and 145,043, respectively, which is a 3.0-fold and 5.3-fold increase over the Pfs230D1/Alhydrogel GMT of 27,318. The titers further increase on day 144 to 6.2-fold for Pfs230D1-E2p/Alhydrogel (GMT 56,892) and 7.6-fold for Pfs230D1-E2p/addaS03 (GMT 70,061) when compared to Pfs230D1/Alhydrogel (GMT 9204) (Fig. 3b, c, e). At the last time point on day 144/145, the antibody titers are also significantly higher for either nanoparticle vaccine (Pfs230D1-E2p/Alhydrogel or Pfs230D1-E2p/AddS03) than Pfs230D1/Alhydrogel (*p*-value = 0.0161, *p*-value = 0.0028, respectively) (Fig. 3e).

In addition to the Pfs230D1 titers, the E2p response on day 35 and day 144/145 was evaluated alongside the Pfs230D1 response at a dilution of 1:25,000 in ELISA (Supplementary Fig. S2). Pfs230D1/Alhydrogel and Pfs230D1/AddaS03 did not produce an E2p-specific antibody response on day 35, while Pfs230D1/Alhydrogel had a response to Pfs230D1 (Supplementary Fig. S2a). Pfs230D1-E2p/Alhydrogel and Pfs230D1-E2p/AddaS03 resulted in a measurable response to E2p on day 35, and this relative response to E2p was much lower than the response to Pfs230D1 at the same dilution (Supplementary Fig. S2a). A similar trend was seen on day 144/145 for Pfs230D1/Alhydrogel, Pfs230D1-E2p/Alhydrogel, and Pfs230D1-E2p/AddaS03 (Supplementary Fig. S2b).

### Pfs230D1-E2p nanoparticle vaccines produce potent and long-lasting transmission-blocking activity

**Fig. 4:**
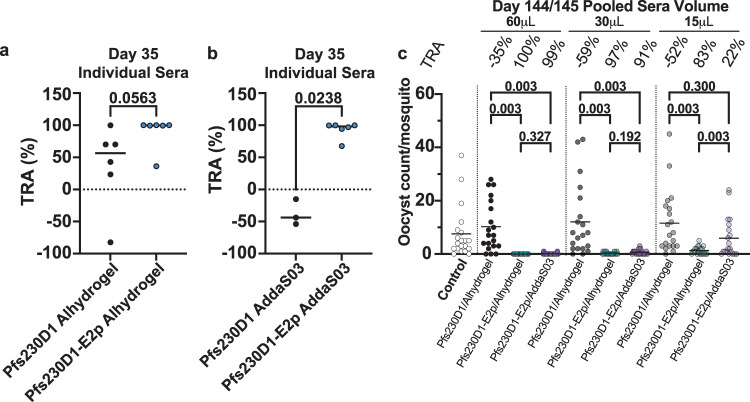
Fusion of Pfs230D1 to E2p enhances the potency and durability of transmission-reducing activity (TRA). **a** Transmission-reducing activity by SMFA of sera from animals immunized with Pfs230D1/Alhydrogel (black circles) and Pfs230D1-E2p/Alhydrogel (blue circles) at a dilution of 60 μL heat-inactivated immunized rabbit serum in a total volume of 260 μL. Each circle represents the average of 20 individually dissected mosquito midguts. **b** Transmission-reducing activity by SMFA of sera from animals immunized with Pfs230D1/AddaS03 (black circles) and Pfs230D1-E2p/AddaS03 (blue circles) at a dilution of 60 μL heat-inactivated immunized rabbit serum in a total volume of 260 μL. Each circle represents the average of 20 individually dissected mosquito midguts. **c** Oocyst counts per mosquito and transmission-reducing activity by SMFA of pooled day 144/145 sera from animals immunized with Pfs230D1/Alhydrogel (black circles), Pfs230D1-E2p/Alhydrogel (teal circles) and Pfs230D1-E2p/AddaS03 (purple circles) at three dilutions of sera of 60 μL, 30 μL, or 15 μL in a total volume of 260 μL. A malaria naïve human sera sample was used as the negative control (white circles). All SMFAs were performed with a feeder total volume of 260 μL that includes 100 μL packed RBCs. Each circle is the total number of oocysts in a single mosquito midgut. Twenty mosquito midguts were dissected for each sample tested. For the graphs in (**a**) and (**b**), the median for each group is shown as a solid line, and the *p*-values were determined using a Mann–Whitney test. For the graph in (**c**), the mean for each group is shown as a solid line, and the *p*-values were determined using a zero-inflated negative binomial model (ZINB model).

The standard membrane feeding assay (SMFA) is the gold-standard method for evaluating the transmission of parasites to mosquitos through a blood meal and measures the transmission-reducing activity (TRA) of serum. We compared the TRA elicited by immunization with Pfs230D1/Alhydrogel and Pfs230D1-E2p/Alhydrogel by analyzing heat-inactivated serum collected on day 35 at a dilution of 60 μL heat-inactivated immunized rabbit serum in a total volume of 260 μL (Fig. 4a). Due to the high variability of TRA in sera from the Pfs230D1/Alhydrogel group (from −82.3 to 99.8%, median of 56.55%), the difference between the two groups did not reach a statistical significance (*p* = 0.0563), but 5/6 animals immunized with Pfs230D1-E2p/Alhydrogel showed >99.5% TRA (median of 99.75%). The one animal in this group that had lower inhibition (36.2% TRA) also had much lower Pfs230D1-binding antibody titers than the other animals on day 35. The serum from animals immunized with Pfs230D1/AddaS03 and Pfs230D1-E2p/AddaS03 were also analyzed for TRA (Fig. 4b). All three sera from animals immunized with Pfs230D1/AddaS03 showed low TRA. In contrast, 5/6 animals immunized with Pfs230D1-E2p/AddaS03 had >96.9% TRA and the sixth animal had 67.7% TRA. The TRA for the Pfs230D1-E2p/AddaS03 group was significantly higher than the TRA for the Pfs230D1/AddaS03 group (*p* = 0.0238). Animals immunized with Pfs230D1-E2p/AddaS03 had significantly higher TRA than those immunized with Pfs230D1/AddaS03 (*p* = 0.0238).

The durability of the response was evaluated by SMFA using pooled day 144/145 sera from animals immunized with Pfs230D1/Alhydrogel, Pfs230D1-E2p/Alhydrogel, and Pfs230D1-E2p /AddaS03 at three dilutions of sera of 60 μL, 30 μL, or 15 μL in a total volume of 260 μL (Fig. 4c). The pooled sera for Pfs230D1/Alhydrogel had no TRA at any dilution tested including the highest concentration of immunized sera tested where activity was observed at day 35 with some of the individual serum. This indicates the monomer does not elicit a durable transmission-blocking antibody response. In contrast, both immunizations with nanoparticle retained potent TRA (>91%) for the 60 μL and 30 μL immunized rabbit sera concentrations at day 144/145. These results indicate E2p nanoparticle presentation of Pfs230D1 in either adjuvant results in a potent and durable transmission-blocking antibody response that is significantly enhanced over the monomer Pfs230D1.

## Discussion

Malaria continues to be a major burden on developing countries around the world, and new approaches for control are desperately needed^4^. The COVID-19 pandemic has only increased this need as, according to the World Health Organization, disruptions to service have likely led to some of the increase in total case numbers and deaths seen in 2021^4^. Developing new cost-effective and efficacious tools is vital to continue the progress seen in previous years in the effort to eradicate this disease. One major effort in the eradication of malaria is the development of potent and durable transmission-blocking vaccines. Pfs230 is the most advanced TBV candidate and Pfs230D1-EPA^22–26^ is currently in clinical trials (NCT02334462, NCT02942277, NCT03917654, NCT05135273). TBV candidates other than Pfs230^3,6–13,15–25,27–29,62,63,65–76^ under clinical evaluation include Pfs25^3,62–64^ and Pfs48/45^3,62,72–75^, and other antigens have been identified and are in development^35,77^.

A single-component nanoparticle system with high potency and durability has the potential to reduce the cost and complexity of production for vaccines. The 60-copy Pfs230D1-E2p nanoparticle developed here can be purified using a tag-less simplified purification scheme, is homogenous and stable, and can be recognized by a D1-specific transmission-blocking human antibody. Critically, immunization of rabbits with two low (1 µg) doses of Pfs230D1-E2p formulated in adjuvants suitable for translation to humans produced antibodies that had high TRA. Immunization with the nanoparticle also produced a potent long-lived antibody response specific for Pfs230D1 above levels seen for the Pfs230D1 monomer formulated in Alhydrogel, and this high TRA was maintained as far as 15.5 weeks post-immunization, which was the endpoint of the study. While the Pfs230D1 monomer formulated in Alhydrogel elicited a Pfs230D1-specific antibody response, the Pfs230D1 monomer formulated in AddaS03 did not. This is not surprising as small proteins and peptides are generally less immunogenic than larger proteins or nanoparticles. Although AddaS03 was a poor adjuvant for the monomer, the nanoparticle formulated in AddaS03 produced a robust Pfs230D1-specific antibody response. This emphasizes the importance of nanoparticle display to improve immunogenicity in multiple adjuvants.

Pfs230D1-E2p immunization studies in rabbits were conducted using both Alhydrogel and AddaS03, which is a research-grade equivalent of AS03, as adjuvants. These adjuvants were chosen for this study because both adjuvants have been proven safe for use in humans. Both formulations of Pfs230D1-E2p proved effective in rabbits. Alhydrogel has been shown to produce primarily an IgG1 response in humans, and AS03 has been shown to produce a more balanced IgG subtype response^57–61^. D1-specific antibodies require complement for function, and IgG1 and IgG3 are the main subtypes in humans that fix complement^69,70^. Previous studies with the Pfs230D1-EPA conjugate formulated in Alhydrogel showed an antibody response in *Rhesus macaque* that was primarily composed of IgG1 subclass antibodies that correlated with high TRA^25^. Another recent study of the Pfs230D1-EPA conjugate adjuvanted in ALFQ (Army Liposome Formulation containing QS21 saponin) showed that this formulation elicited a more widespread IgG subclass response in *Rhesus macaque*^68^. After 2 doses of the Pfs230D1-EPA/ALFQ formulation, the antibody response was dominated by IgG1 and IgG3, but this changed after the third dose^68^. After three doses, high levels of IgG2 subclass antibodies could be observed^68^. More importantly, the IgG1 response rapidly diminished while the IgG2 maintained high titers over the course of the study, with the endpoint being 600 days post the first immunization^68^. The IgG2 antibodies also had high TRA at day 600, showing that other IgG subclasses outside of IgG1 can have an effect on TRA^68^. These studies show that adjuvant selection may be critical in developing the desired antibody subclass response, and the demonstration here that the nanoparticles are versatile and can be successfully used with different adjuvants raises the possibility for the further development and optimization of vaccine dose and formulation.

Another important consideration for vaccines is the ability of the pathogen to evade antibody responses^78,79^. A recent study identified the human epitope map after immunization with the Pfs230D1-EPA conjugate formulated in Alhydrogel and AS01^5^. Analysis of the antibodies raised by vaccination showed that antibodies with high TRA all bound to one face of Pfs230D1, and the antibodies with low TRA bound to the opposite face^5^. The study also identified 10 polymorphisms in Pfs230D1 using 2512 sequences from MalariaGen.net^5^. Of the 10 polymorphisms, only G605S/R and D714N, present in 91.49% and 0.04% of sequences, respectively, had an effect on the binding of antibodies with high TRA^5^. It will be important to incorporate these polymorphisms into our Pfs230D1-E2p nanoparticle in future studies looking at the response to the new constructs in isolation and in combination with our current construct.

The data presented demonstrate a highly improved potency and durability upon immunization of the nanoparticle over the monomer in two clinically relevant adjuvants that can be translated to use in humans. However, the study is limited in that it was performed once in rabbits and not developed to unequivocally identify a single adjuvant for future use. Another limitation is that molecular weight markers for the gel filtration profiles are not provided, however, the multi-angle static light scattering and small angle X-ray scattering results provide robust biophysical measurements of particle size and shape far more accurate than estimation of mass using molecular weight markers. In addition, rabbits do not possess diverse IgG subclasses, and the study is limited in that the antibody subclasses elicited by the particle versus the monomer cannot be addressed. Future studies to improve the nanoparticles using structure-based design^14,56,80,81^ and to examine the response and antibody subtypes elicited in non-human primates are supported by the promising data in preclinical rodent studies presented here.

This study is a proof of concept that a single-component nanoparticle TBV can be produced and elicit potent and durable responses in preclinical animal studies. The data suggest that Pfs230D1-E2p has the potential to improve upon the current-generation transmission-blocking vaccines. The ease of production of the single-component self-assembling Pfs230D1-fused nanoparticle and the demonstration that two diverse adjuvants are suitable to produce protective responses form a strong foundation for the development of a cost-effective, potent, and durable TBV. This prototype single-component Pfs230D1 TBV has the potential to reduce malaria transmission in endemic areas and could play a role in malaria control and eradication efforts.

## Methods

### Generation of expression plasmids

The sequence for *Plasmodium falciparum* Pfs230D1 (3D7 accession number XP_001349600.1) was synthesized and subcloned into pHLSec by Genscript. The domain boundaries used were previously described and include amino acids 542–588 of the N-terminal region and amino acids 589–736 that define D1^7,24,25,68^. The sequence of Pfs230D1 was also subcloned by Genscript into a modified pHLSec plasmid containing the sequence for amino acids 185–426 of E2p from *G. stearothermophilus* (PDB 1B5S_A). The plasmid also contains a 15-amino acid linker (GGGGS)_3_ between Pfs230D1 and E2p. This plasmid allows for the expression of a fusion protein consisting of Pfs230D1-linker-E2p. The sequence for *Plasmodium falciparum* Pfs25 (PF3D7_1031000) was synthesized and subcloned into pHLSec by Genscript. The domain boundaries used were previously described. The LMIV-01 sequence was isolated from a human subject immunized with Pfs230D1-EPA/Alhydrogel. The variable regions for the heavy and light chains of LMIV-01 were synthesized and subcloned by Genscript into modified pHLSec plasmids containing the constant regions for human IgG1 heavy and light chains, respectively. The noNAG-RBD-E2p pHLSec plasmid was also produced by Genscript as previously described^56^.

### Expression and purification of Pfs230D1, Pfs230D1-E2p, Pfs25, LMIV-01 IgG, noNAG-RBD-E2p

Pfs230D1, Pfs230D1-E2p, Pfs25, LMIV-01, and noNAG-RBD-E2p were transiently expressed in Expi293F cells (Life Technologies ThermoFisher Scientific) using the Expi293 expression system (Life Technologies ThermoFisher Scientific). On day 4 post-transfection, the culture supernatant was harvested by centrifugation.

The Pfs230D1 and Pfs25 were then purified using Ni Sepharose excel resin (Cytiva). The resin was equilibrated with 20 mM sodium phosphate buffer, pH 7.4 and 100 mM NaCl. The Pfs230D1 was then added to the resin. The resin was then washed with 20 mM sodium phosphate buffer pH 7.4, 100 mM NaCl and 10 mM Imidazole. The Pfs230-D1 was then eluted from the resin with 20 mM sodium phosphate buffer, pH 7.4, 100 mM NaCl and 500 mM Imidazole. The eluted protein was then concentrated using a 3 kDa AMICON spin filter (EMD Millipore). The concentrated protein was further purified using a Superdex 75 Increase 10/300 GL column (Cytiva) and phosphate-buffered saline (PBS).

The Pfs230D1-E2p supernatant was concentrated using a 50 kDa AMICON spin filter (EMD Millipore). The concentrated protein was further purified using a Sepax SRT SEC-1000 column (Sepax Technologies) and either PBS for use in functional assays and immunization or 10 mM HEPES, pH 7.4, 100 mM NaCl for Negative Stain.

LMIV-01 IgG supernatant was purified using protein A resin (GoldBio). Protein A resin was equilibrated with Protein A IgG binding buffer (ThermoFisher Scientific). LMIV-01 supernatant was diluted 1:2 with Protein A IgG binding buffer and then added to the resin. The resin was then washed with Protein A IgG binding buffer. LMIV-01 was then eluted off the column into PBS with Protein A IgG elution buffer (ThermoFisher Scientific). The eluted protein was then concentrated using a 50 kDa AMICON spin filter (EMD Millipore). The concentrated protein was further purified using a Superdex 200 Increase 10/300 GL column (Cytiva) and Phosphate buffered saline (PBS).

The noNAG-RBD-E2p supernatant was diluted 1:2 with 50 mM Tris pH 8.0 and purified using Q sepharose Fast Flow resin (Cytiva). The resin was equilibrated with 50 mM Tris pH 8.0, 50 mM NaCl before adding the diluted supernatant. The supernatant was then added to the resin by gravity flow. The resin was washed with 50 mM Tris pH 8.0, 50 mM NaCl and then the protein was eluted with 50 mM Tris, 750 mM NaCl. The elution was concentrated using a 50 kDa AMICON spin filter (EMD Millipore). The concentrated protein was further purified using a Superose 6 Increase 10/300 GL column (Cytiva) and phosphate-buffered saline (PBS).

### Mass spectrometry protein identification

Each Coomassie-stained band on the SDS-PAGE gel was excised, then processed with a standard in-gel digestion protocol. The trypsin-digested peptide samples were analyzed and subjected to the LC-MS. The sample was separated using a PepMap C18 analytical column (Thermo Scientific, 75 µm diameter, 25 cm length, 2 µm particles) using a 60-min acetonitrile gradient at the flow rate of 200 nL/min. The MS data acquisition was carried out in a data-dependent acquisition mode using Orbitrap Fusion™ Lumos™ Mass Spectrometer (Thermo Scientific). The data were analyzed using Proteome Discoverer (version 2.4) against the concatenated databases of the recombinant protein, Pfs230D1-E2p, human proteins from uniprot.org (https://www.uniprot.org/taxonomy/9606), and the cRAP commonly contaminating protein database from theGPM.org (https://thegpm.org/crap/).

### Negative-stain electron microscopy of Pfs230D1-E2p and 2D classification

Purified Pfs230D1-E2p complexes were adsorbed on a glow discharged 300 mesh carbon-coated copper grids (EMD Science) at a concentration of 0.01 mg/mL for 30 s followed by methylamine tungstate (NanoW ®- Nanoprobes Inc.) staining. Raw Micrographs were recorded in a Thermo Scientific Tecnai T20 microscope equipped with a charge-coupled device (CCD) camera. Particles were auto-picked using Gautomatch (http://www.mrc-lmb.cam.ac.uk/kzhang/), and 2D classes were generated using RELION 3.0^82^.

### Stability analysis of Pfs230D1-E2p

Pfs230D1-E2p was purified as described above in 10 mM HEPES, pH 7.4, 100 mM NaCl buffer. Each biological replicate was split evenly between a time 0 and treatment condition sample. The time 0 sample was then purified using a Sepax SRT SEC-1000 column (Sepax Technologies), and the amount of protein in the peak was determined using the Evaluation suite of UNICORN 7.3 software (General Electric Company). The treatment sample after treatment was purified using a Sepax SRT SEC-1000 column (Sepax Technologies), and the amount of protein in the peak was determined using the Evaluation suite of UNICORN 7.3 software (General Electric Company). The Evaluation program determines the concentration of the protein in a given peak using the formula Concentration [mg/mL] = A/(d × 1000 × extinction coefficient), where A = average peak absorbance (Area/volume [mAu]) and d = UV cell path length [cm]. The percent recovered protein was analyzed using GraphPad Prism 9 for MacOS. The freeze/thaw treatment was conducted by placing the sample in a −80 °C freezer and then thawing it the next day and purifying it as described. The 4 °C treatment was conducted by placing the sample in a 4 °C refrigerator for 24 h and purifying it as described. The room temperature treatment was conducted by placing the sample on the lab bench for 24 h and purifying it as described.

### LMIV-01 binding to Pfs230D1 and Pfs230D1-E2p

Nunc MaxiSorp ELISA plates (ThermoFisher Scientific) were coated with 0.02 mg/mL of Pfs230D1, Pfs230D1-E2p, and Pfs25 in Carbonate buffer, pH 9.6 overnight at 4 °C. The plates were then washed three times with PBS and 0.05% Tween 20 (PBST). The plates were then blocked with 2% Bovine Serum Albumin (BSA) (Sigma) in PBST for 1 h at room temperature. The plates were then washed three times with PBST. The plates were then incubated with 250 ng/mL of LMIV-01 in 2%BSA, PBST for 1 h at room temperature. The plates were then washed three times with PBST. The plates were then incubated with a 1:5000 dilution of anti-human horseradish peroxidase-conjugated secondary antibody (Jackson ImmunoResearch Laboratories) for 1 h at room temperature. The plates were then washed three times with PBST. The plates were then incubated with 3,3′,5,5′-Tetramethylbenzidine (TMB) Liquid Substrate System for ELISA (Millipore Sigma Aldrich). The reaction was stopped by the addition of 2 M sulfuric acid to a final concentration of 1 M. The plates were read with a Synergy H1 plate reader and 5gen v3.08 software. The data were graphed and analyzed using GraphPad Prism 9 for MacOS.

### Size-exclusion chromatography small angle X-ray scattering (SEC-SAXS) and multi-angle light scattering (MALS)

SEC-SAXS and MALS were conducted at SIBYLS at beamline 12.3.1^83–86^. In brief, samples were injected into a size-exclusion chromatography column and a post column splitter directed the elution to the MALS system and the SAXS system. The MALS system consisted of an Agilent 1260 series multiple wavelength detector, a Wyatt Dawn Helos multi-angle light scattering detector, a Wyatt DyanPro Titan quasi-elastic light scattering detector, and a Wyatt Optilab rEX refractometer. The MALS data were analyzed using Wyatt Astra 6 software. The data for SAXS were collected as the protein came off the column with a λ = 1.03 Å incident light at a sample-to-detector distance of 1.5 m. A series of 3-s exposures were collected for each frame over the course of 40 min. SAXS data were analyzed using the ATSAS 3.0.5.2 software suite for macOS^87^.

### Immunization of rabbits with Pfs230D1 and Pfs230D1-E2p

All rabbit studies were performed in an AAALAC-accredited facility under the guidelines and approval of the Institutional Animal Care and Use Committee (IACUC) at the National Institutes of Health. A dose of 1 μg of Pfs230D1 and Pfs230D1-E2p was added to 1 mg/mL Alhydrogel (Invivogen), final dose of 500 μg alum per injection, in DPBS and incubated rotating for 1 h at room temperature. A dose of 1 μg of Pfs230D1 and Pfs230D1-E2p was added to a 1:1 volume of AddaS03 (Invivogen) and mixed by pipetting up and down 10 times. All formulations were kept at room temperature in the time between formulation and injection into rabbits. Female rabbits approximately 11 weeks of age and weighing between 2.2 and 2.6 kg were obtained from Charles River Laboratories. On day 0 and day 21 of the study, rabbits were immunized subcutaneously in one site in the dorsal area with a volume of 500 μL containing a dose of 1 µg of antigen in either Alhydrogel or AddaS03 formulated as described above. Rabbits immunized with Pfs230D1/AddaS03 were bled for sera on days 0, 14, and 35 of the study. All other study groups were bled for sera on days 0, 14, 35, 63, 91, 119, and either 144 or 145.

### Determination of Pfs230D1-specific titers in rabbit sera samples

Antibody titers were determined by ELISA with the use of a standard curve. The standard curve consisted of rabbit sera with Pfs230-D1 antibodies. The sera from each animal at each time point was diluted to be within the linear range of the standard curve when incubated with TMB for 5 min. Nunc MaxiSorp ELISA plates (ThermoFisher Scientific) were coated with 0.02 mg/mL of Pfs230D1 in Carbonate buffer, pH 9.6 overnight at 4 °C. The plates were then washed three times with PBS and 0.05% Tween 20 (PBST). The plates were then blocked with 2% Bovine Serum Albumin (BSA) (Sigma) in PBST for 1 h at room temperature. The plates were then washed three times with PBST. The plates were then incubated with diluted poly sera in 2% BSA, PBST for 1 h at room temperature. The plates were then washed three times with PBST. The plates were then incubated with a 1:5000 dilution of anti-rabbit HRP conjugated secondary antibody (Bethyl Laboratories) for 1 h at room temperature. The plates were then washed three times with PBST. The plates were then incubated with 3,3′,5,5′-Tetramethylbenzidine (TMB) Liquid Substrate System for ELISA (Millipore Sigma Aldrich) for 5 min and the reaction stopped by the addition of 2 M sulfuric acid to a final concentration of 1 M. The plates were read with a Synergy H1 plate reader and 5gen v3.08 software. The data were graphed and analyzed using GraphPad Prism 9 for MacOS.

### Determination of E2p-specific antibody response in rabbit sera samples

The sera from each animal at each time point was diluted 1:25,000 with 2% BSA in PBST. Nunc MaxiSorp ELISA plates (ThermoFisher Scientific) were coated with 0.01 mg/mL of either Pfs230D1 or noNAG-RBD-E2p in Carbonate buffer, pH 9.6 overnight at 4 °C. The plates were then washed three times with PBS and 0.05% Tween 20 (PBST). The plates were then blocked with 2% BSA in PBST for 1 h at room temperature. The plates were then washed three times with PBST. The plates were then incubated with diluted poly sera for 1 h at room temperature. The plates were then washed three times with PBST. The plates were then incubated with a 1:5000 dilution of anti-rabbit HRP conjugated secondary antibody (Bethyl Laboratories) for 1 h at room temperature. The plates were then washed three times with PBST. The plates were then incubated with TMB Liquid Substrate System for ELISA and the reaction stopped by the addition of 2 M sulfuric acid to a final concentration of 1 M. The plates were read with a Synergy H1 plate reader and 5gen v3.08 software. Three technical replicates were run for each sera sample, and the average of the three was plotted for each sample. The data were graphed using GraphPad Prism 9 for MacOS.

### Standard membrane feeding assay

The SMFA was conducted as described previously^88^. In brief, for the SMFA of individual sera samples, 60 μL of heat-inactivated immunized rabbit serum were mixed with ~0.2% of cultured stage V gametocytes (*P. falciparum* NF54 strain) in the presence of human complement (i.e., using non-heat-inactivated human serum collected from malaria naïve US adults) for a total volume of 260 μL (60 μL rabbit serum, 100 μL human serum, 100 μL packed RBCs), and then fed to 3- to 6-day-old female *Anopheles stephensi*. The mosquitoes were maintained for 8 days and then dissected to count the number of oocysts per midgut in 20 blood-fed mosquitoes. For the SMFA of the pooled sera, either 60 μL immunized sera, a mixture of 30 μL of an immunized serum with 30 μL of non-immunized rabbit serum, or a mixture of 15 μL of an immunized serum with 45 μL of non-immunized rabbit serum were mixed with 100 μL of human serum and 100 μL of packed RBC to feed mosquitoes.

### Reporting summary

Further information on research design is available in the Nature Research Reporting Summary linked to this article.

### Supplementary information

Supplemental Material

### Source data

Source data

REPORTING SUMMARY

## Data Availability

All data needed to evaluate the conclusions in the paper are present in the paper and/or Supplementary Materials. Plasmids can be provided by N.H.T. pending scientific review and a completed material transfer agreement. Requests should be submitted to N.H.T.
