# Supplementary material for: A potent and durable malaria transmission-blocking vaccine designed from a single-component 60-copy Pfs230D1 nanoparticle
Source: NPJ Vaccines. 2023 Aug 18;8:124. doi: 10.1038/s41541-023-00709-8 (PMC10439124; doi:10.1038/s41541-023-00709-8)
Supplement: Supplementary file 1 — Supplemental Material [file 41541_2023_709_MOESM1_ESM.docx]

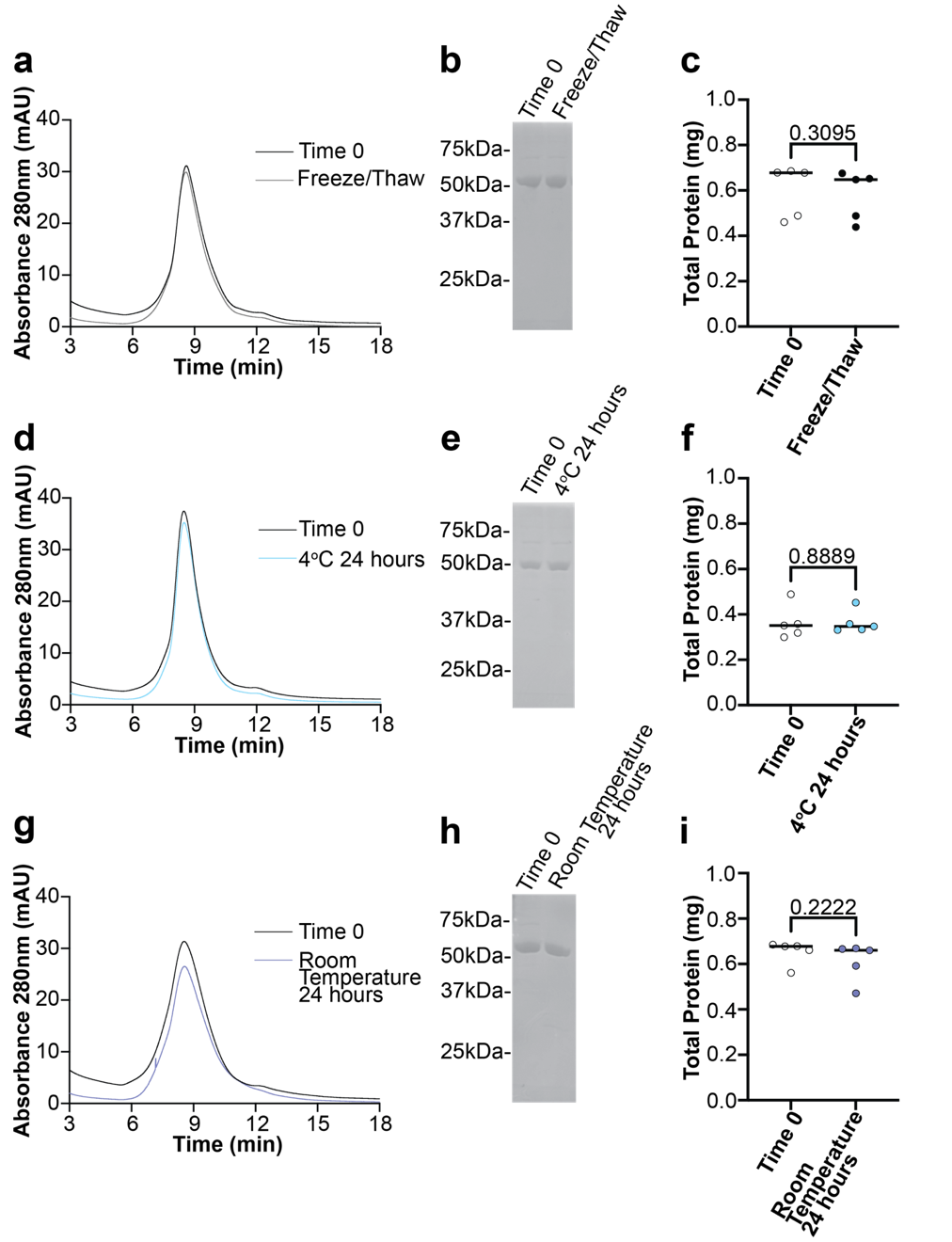


**Supplementary Figure 1. Pfs230D1-E2p nanoparticles are stable under multiple varied conditions.** One representative biological replicate of **a** size exclusion chromatogram, **b** reduced SDS-PAGE gel electrophoresis, and **c** quantified recover after size exclusion chromatography for all individual biological replicates of Pfs230D1-E2p at the time of initial purification (Time 0) and after being frozen and thawed. One representative biological replicate of **d** size exclusion chromatogram, **e** reduced SDS-PAGE gel electrophoresis, and **f** quantified recover after size exclusion chromatography for all individual biological replicates of Pfs230D1-E2p at the time of initial purification (Time 0) and after incubation for 24 hours at 4oC. One representative biological replicate of **g** size exclusion chromatogram, **h** reduced SDS-PAGE gel electrophoresis, and **i** quantified recover after size exclusion chromatography for all individual biological replicates of Pfs230D1-E2p at the time of initial purification (Time 0) and after incubation for 24 hours at room temperature. All graphs in **c, f,** and **i** show the individual biological replicates with the median and p-values which were determined using a Mann-Whitney test.


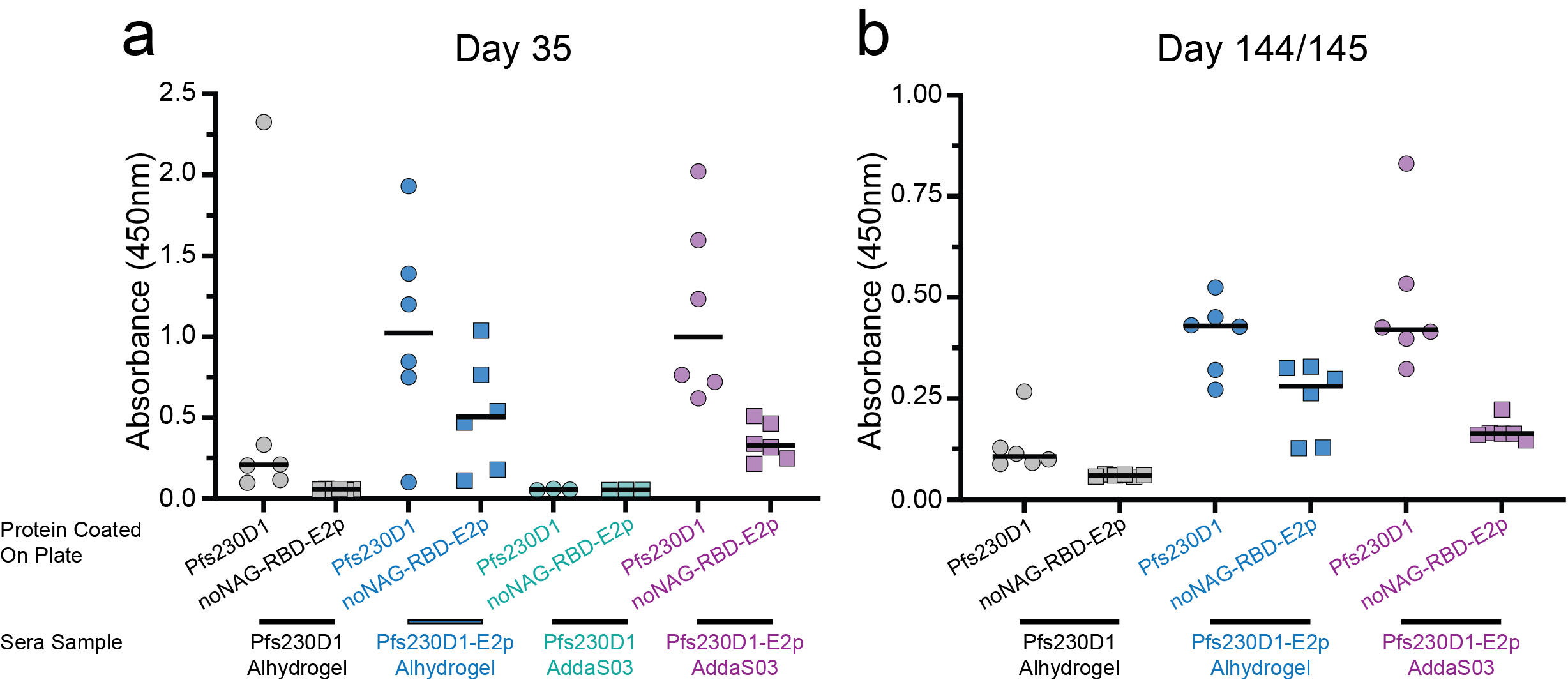


**Supplementary Figure 2. Pfs230D1-E2p induces fewer E2p specific antibodies than Pfs230D1 antibodies.** Pfs230D1 and E2p specific antibody responses at a dilution of 1:25,000 for rabbits immunized with Pfs230D1/Alhydrogel, Pfs230D1-E2p/Alhydrogel, Pfs230D1/AddaS03, and Pfs230D1-E2p/AddaS03 on **a** day 35 and **b** day 144/145. (◯) Pfs230D1 coated on the ELISA plate () noNAG-RBD-E2p coated on the ELISA plate
