# Supplementary material for: A potent and durable malaria transmission-blocking vaccine designed from a single-component 60-copy Pfs230D1 nanoparticle
Source: NPJ Vaccines. 2023 Aug 18;8:124. doi: 10.1038/s41541-023-00709-8 (PMC10439124; doi:10.1038/s41541-023-00709-8)
Supplement: Supplementary file 3 — REPORTING SUMMARY [file 41541_2023_709_MOESM3_ESM.pdf]

nature portfolio

Corresponding author(s): Niraj Tolia

Last updated by author(s): Dec 7, 2020

Reporting Summary

Nature Portfolio wishes to improve the reproducibility of the work that we publish. This form provides structure for consistency and transparency in reporting. For further information on Nature Portfolio policies, see our Editorial Policies and the Editorial Policy Checklist.

Statistics

For all statistical analyses, confirm that the following items are present in the figure legend, table legend, main text, or Methods section.

n/a	Confirmed	
☐	☒	The exact sample size (*n*) for each experimental group/condition, given as a discrete number and unit of measurement
☐	☒	A statement on whether measurements were taken from distinct samples or whether the same sample was measured repeatedly
☐	☒	The statistical test(s) used AND whether they are one- or two-sided<br>*Only common tests should be described solely by name; describe more complex techniques in the Methods section.*
☒	☐	A description of all covariates tested
☒	☐	A description of any assumptions or corrections, such as tests of normality and adjustment for multiple comparisons
☐	☒	A full description of the statistical parameters including central tendency (e.g. means) or other basic estimates (e.g. regression coefficient) AND variation (e.g. standard deviation) or associated estimates of uncertainty (e.g. confidence intervals)
☒	☐	For null hypothesis testing, the test statistic (e.g. F, t, r) with confidence intervals, effect sizes, degrees of freedom and P value noted<br>*Give P values as exact values whenever suitable.*
☒	☐	For Bayesian analysis, information on the choice of priors and Markov chain Monte Carlo settings
☒	☐	For hierarchical and complex designs, identification of the appropriate level for tests and full reporting of outcomes
☒	☐	Estimates of effect sizes (e.g. Cohen's *d*, Pearson's *r*), indicating how they were calculated

Our web collection on statistics for biologists contains articles on many of the points above.

Software and code

Policy information about availability of computer code

Data collection	UNICORN 7.3 was used for the collection and quantification of protein concentration during purification.<br>5gen v3.08 software was used for the collection of ELISA data.
Data analysis	Graphpad Prism 9 for MacOS was used for all analysis of statistical comparisons.<br>Gautomatch and RELION 3.0 were used for the analysis of negative stain Electron Microscopy data.<br>Wyatt Astra 6 software was used for the analysis of MALS data.<br>ATSAS 3.0.5.2 software suite for MacOS was used for the analysis of SEC-SAXS data.

For manuscripts utilizing custom algorithms or software that are central to the research but not yet described in published literature, software must be made available to editors and reviewers. We strongly encourage code deposition in a community repository (e.g. GitHub). See the Nature Portfolio guidelines for submitting code & software for further information.

Data

Policy information about availability of data

All manuscripts must include a data availability statement. This statement should provide the following information, where applicable:

- Accession codes, unique identifiers, or web links for publicly available datasets
- A description of any restrictions on data availability
- For clinical datasets or third party data, please ensure that the statement adheres to our policy

All data needed to evaluate the conclusions in the paper are present in the paper and/or Supplementary Materials.

Human research participants

Policy information about studies involving human research participants and Sex and Gender in Research.

Reporting on sex and gender	Not applicable
Population characteristics	Not applicable
Recruitment	Not applicable
Ethics oversight	Not applicable

Note that full information on the approval of the study protocol must also be provided in the manuscript.

Field-specific reporting

Please select the one below that is the best fit for your research. If you are not sure, read the appropriate sections before making your selection.

☒ Life sciences ☐ Behavioural & social sciences ☐ Ecological, evolutionary & environmental sciences

For a reference copy of the document with all sections, see nature.com/documents/nr-reporting-summary-flat.pdf

Life sciences study design

All studies must disclose on these points even when the disclosure is negative.

Sample size	Not applicable to this study design
Data exclusions	No data was excluded from this manuscript
Replication	For the analysis of antibody titer, each individual rabbit was treated as a biological replicate and each biological replicate had three technical replicates. The three technical replicates were averaged to produce the final biological replicate used in analysis.<br>For the analysis of stability, five biological replicates were conducted for each condition.<br>For the SMFA of individual rabbit sera, each rabbit was treated as a biological replicate.
Randomization	Individual samples and pooled samples for SMFA were randomized before analysis.
Blinding	Individual samples and pooled samples for SMFA were blinded after randomization before analysis. The individual preforming the SMFA had no knowledge of the identifications of the samples before the experiment was conducted.

Reporting for specific materials, systems and methods

We require information from authors about some types of materials, experimental systems and methods used in many studies. Here, indicate whether each material, system or method listed is relevant to your study. If you are not sure if a list item applies to your research, read the appropriate section before selecting a response.

Materials & experimental systems

n/a	Involved in the study	
☐	☒	Antibodies
☐	☒	Eukaryotic cell lines
☒	☐	Palaeontology and archaeology
☐	☒	Animals and other organisms
☒	☐	Clinical data
☒	☐	Dual use research of concern

Methods

n/a	Involved in the study	
☒	☐	ChIP-seq
☒	☐	Flow cytometry
☒	☐	MRI-based neuroimaging

Antibodies

Antibodies used	LMIV-01 was produced in Expi239F-cells in house for use in ELISA experiments to assess the nanoparticle along with a Peroxidase AffiniPure Goat anti-Human IfG, Fcy fragment specific secondary antibody from Jackson ImmunoResearch Laboratories Inc catalog # 109-035-098, lot # 141861.<br>Goat x-Rabbit IgG-Fc Fragment HRP conjugated antibody from Bethyl laboratories catalog # A-120-111P, lot # 31 was used for all ELISA experiments to determine antibody titers.<br>Rabbit poly-sera against Pfs230D1 and Pfs230D1-E2p was generated in house.
Validation	LMIV-01 was validated in Coelho, C. H. et al. A human monoclonal antibody blocks malaria transmission and defines a highly conserved neutralizing epitope on gametes. Nat Commun 12, 1750 (2021). The secondary antibodies from Jackson ImmunoResearch Laboratories Inc and Bethyl laboratories had been validated for use by the companies.

Eukaryotic cell lines

Policy information about cell lines and Sex and Gender in Research

Cell line source(s)	Expi293F cells were obtained from Life Technologies catalog # A14527.
Authentication	Cell line was obtained from a commercial source.
Mycoplasma contamination	Not tested as the cell line was obtained from a commercial source.
Commonly misidentified lines<br>(See ICLAC register)	not applicable

Animals and other research organisms

Policy information about studies involving animals; ARRIVE guidelines recommended for reporting animal research, and Sex and Gender in Research

Laboratory animals	Female rabbits approximately 11 weeks and weighing between 2.2 and 2.6kg were obtained from Charles River Laboratories.<br>3-6-day old female Anopheles stephensi mosquitoes were grown in our insectory
Wild animals	not applicable
Reporting on sex	Sex was not considered in this study as only female rabbits and mosquitoes were used.
Field-collected samples	not applicable
Ethics oversight	Rabbit studies were performed in an AAALAC-accredited facility under the guidelines and approval of the Institutional Animal Care and Use Committee (IACUC) at the National Institutes of Health

Note that full information on the approval of the study protocol must also be provided in the manuscript.
